# A case of spontaneous avulsion of primary pterygium analyzed with anterior segment optical coherence tomography

**DOI:** 10.1016/j.ajoc.2022.101438

**Published:** 2022-02-19

**Authors:** Takayuki Tanaka, Satoru Kase, Susumu Ishida

**Affiliations:** Department of Ophthalmology, Faculty of Medicine and Graduate School of Medicine, Hokkaido University, Sapporo, Japan

**Keywords:** Pterygium, Spheroidal degeneration, Spontaneous avulsion, Anterior segment optical coherence tomography, AS-OCT, Anterior segment optical coherence tomography

## Abstract

**Purpose:**

This study reports a case of the spontaneous avulsion of primary pterygium with anterior segment optical coherence tomography (AS-OCT) findings.

**Observation:**

A 72-year-old woman complained of acute pain of the left eye. Primary pterygia were noted in both eyes on the initial examination 4 months ago. Slit-lamp microscopy revealed a nasal corneal epithelial defect, and the rolled elevated lesion in the corneal limbus of the left eye. She was diagnosed with a spontaneous avulsion of the corneal pterygium head. Then the avulsed pterygium head slowly recurred. The pterygium head of the fellow eye had a yellow-whitish elevated lesion beneath the epithelium with poor vascularity. AS-OCT revealed hyper-reflective foci beneath the epithelium corresponding to the yellow-whitish elevated lesion.

**Conclusions/importance:**

The present case revealed the spontaneous avulsion of the pterygium head leading to the corneal epithelial defects and ocular pain, while the pterygium head of the fellow eye showed subepithelial hyper-reflective foci suspicious of spheroidal degeneration on AS-OCT. In this case, the cause of spontaneous avulsion of the pterygium head might be potentially weak adhesion to the cornea due to spheroidal degeneration.

**Precis:**

This case is a primary pterygium leading to spontaneous avulsion, in which hyper-reflective foci were noted in OCT.

## 1 Introduction

Pterygium, a common ocular surface disorder, is a triangular fibrovascular proliferation that usually extends from the nasal conjunctiva onto the cornea. The significant environmental risk factors of development of pterygium are long-term exposure to ultraviolet radiation from the sunlight, and to dry and dusty environments.^1^ Bowman's layer breakdown associated with the pterygium extension allows the pterygium head to adhere to the superficial stroma, which in turn creates the with-the-rule astigmatism due to a localized flattening of the corneal curvature.

Generally, it is unlikely that the pterygia spontaneously regress or avulse; therefore, surgical removal is required when it clinically worsens, such as visual impairment, significant discomfort, or esthetic concerns.

Previously, Cook and Dapling first presented a case of pterygium showing a spontaneous resolution by auto-avulsion in 1994.^2^ Later, Son et al. reported a new case of an auto-avulsion and resolution of the pterygium in 2015.^3^ In both cases, although there was no major trauma or injury at the time of onset, but a minor local injury or tensional forces were considered to result in the avulsion.^2^^,^^3^ We herein report the third case of spontaneous avulsion of the pterygium head in an elderly Japanese woman in the literature. Additionally, we exhibited characteristic anterior segment-optical coherence tomography (AS-OCT) findings and considered the pathophysiological findings. The authors confirmed that a statement of consent to publish this case including the images was gathered from the patient.

## 2 Case description

A 72-year-old Japanese woman was referred to our department as a first visit because of a fundus examination for diabetic retinopathy. She had a medical history of diabetes, appendicitis, breast cancer, varix of the lower extremity, pancreas cancer, and bilateral leg cellulitis. The best-corrected visual acuity was 20/16 × Sph+0.50D: Cyl −1.00D Ax100° and 20/16 × Sph+0.25D: Cyl −1.25D Ax85° in the right eye OD and left eye OS, respectively. As shown, the refractive values were against-the-rule astigmatism OU. However, corneal astigmatism obtained by auto keratometer (Tomey RT-7000, Tomey Corp, Tokyo, Japan) were −0.62D Ax123°OD and −0.50D Ax60°OS, indicating oblique astigmatism OU. Intraocular pressure was normal OU. Slit-lamp examination demonstrated similar primary pterygia in the nasal corneal limbus OU. Fundus examination revealed no diabetic retinopathy OU.

**Fig. 1:**
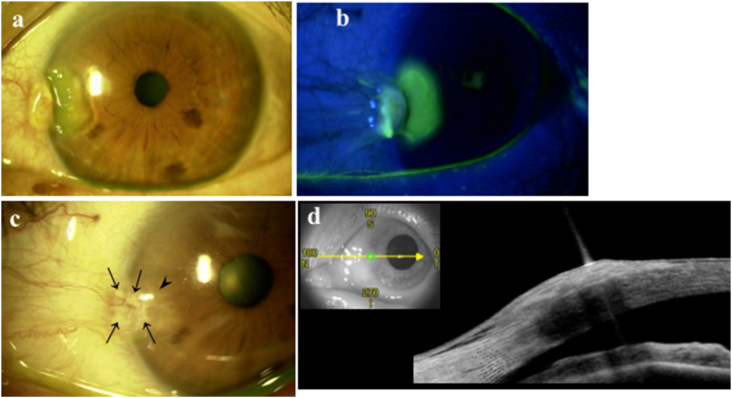
Slit-lamp examination, with fluorescein dye, and AS-OCT findings of the left eye. (a) The round corneal epithelial defect exhibited at the nasal aspect of the cornea and the rolled elevated lesion in the corneal limbus at the second visit when the pain occurred. (b) The round corneal epithelial defect showed a positive sodium fluorescein dye staining at the second visit. (c) Four months later, the pterygium slowly recurred (arrowhead), where a slight yellow-whitish lesion within the recurred pterygium head was observed (arrows). The yellow-whitish lesions were scattered within the pterygium. (d) AS-OCT revealed diffuse thickening with moderate-to-high reflectivity beneath the epithelium 4 months later. However, the hyperreflective foci were no longer identifiable because the yellow-whitish lesions were scattered within the pterygium tissue.

**Fig. 2:**
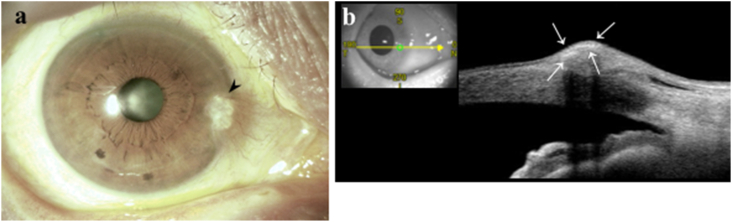
Slit-lamp examination (**a)** and AS-OCT (**b**) findings of the right eye 4 months later. (a) The pterygium head had a yellow-whitish elevated lesion beneath the epithelium with poor vascularity (arrow head). (b) AS-OCT revealed hyper-reflective foci beneath the epithelium corresponding to the yellow-whitish elevated lesion (arrows). . (For interpretation of the references to colour in this figure legend, the reader is referred to the Web version of this article.)

Four months later, she complained of acute ocular pain OS, and was referred to our department again because the pain didn't improve due to unknown causes. She had no history of prior ocular trauma. Slit-lamp microscopy revealed a round corneal epithelial defect at the nasal aspect of the cornea with positive fluorescein sodium dye staining, together with the rolled elevated lesion in the corneal limbus OS (Fig. 1 a, b). She was diagnosed with a spontaneous avulsion of the pterygium head because she had primary pterygia OU on the initial examination, and then an ofloxacin ointment was given three times a day. After the treatment, the pain disappeared the next day. Three days after the treatment, the corneal epithelial defect was completely epithelialized. The pterygium head of the fellow eye had a yellow-whitish elevated lesion beneath the epithelium with poor vascularity OD (Fig. 2a, arrowhead). AS-OCT revealed hyper-reflective foci beneath the epithelium corresponding to the yellow-whitish elevated lesion OD (Fig. 2b, arrows). One and a half months later, the corneal astigmatism was −0.18D Ax 89° OS, showing the astigmatic shift toward 90° compared to the initial examination. On the other hand, the avulsed pterygium head slowly recurred OS (Fig. 1c, arrowhead), with increased vascularity and a slight yellow-whitish lesion within the recurred pterygial tissue（Fig. 1c, arrows）. AS-OCT revealed diffuse thickening with moderate-to-high reflectivity beneath the epithelium OS (Fig. 1d). Four months later, the corneal astigmatism was −0.37D Ax65° OS, showing the astigmatic shift toward 180° with the recurrence of the pterygium compared to that one and a half months later.

## 3 Discussion

As shown in the present case, a spontaneous avulsion of the pterygium head seems to be a rare phenomenon in primary pterygia. To the best of our knowledge, this case is the third report of a spontaneous avulsion of the pterygium head which had no history of prior trauma or injury of the globe.^2^^,^^3^ Same as the previous report,^3^ our present case also gradually recurred after the spontaneous avulsion of the pterygium head. In addition, the pterygia OU were considered to present with a yellow-whitish elevated lesion beneath the epithelium with poor vascularity, where AS-OCT revealed highly reflective foci before the onset of avulsion OS. Generally, AS-OCT findings of the pterygium demonstrate diffuse thickening with moderate-high reflectivity beneath the epithelium,^4^ whereas it never shows highly reflective banded foci like the present case. The origin of the hyper-reflective foci remains unknown in this case. Recently, we experienced a case of ocular surface squamous neoplasia with histology-proven spheroidal degeneration, the latter of which showed yellowish granules and hyper-reflective foci on slit-lamp examination and AS-OCT, respectively (unpublished data). Therefore, the AS-OCT findings in the present case were considered to be a spheroidal degeneration.

Spheroidal degeneration is observed in the cornea and conjunctiva, which is especially common in people who live in areas with high ultraviolet radiation from the sunlight.^5^ Previous studies have suggested that the source of spheroidal degeneration could be due to the accumulation of plasma proteins, possibly immunoglobulins, and albumins modified by ultraviolet irradiation.^6^ In the cornea, deposits of spheroidal degeneration are generally located in the subepithelial layer, Bowman's layer, and superficial stroma of the cornea.^5^ It causes an irregular surface of the cornea, which leads to epithelial breakdown, resulting in symptoms of pain and photophobia.^7^ In this case, adhesion of the pterygium head to the cornea might have been weak because spheroidal degenerative materials are deposited within the pterygium tissue, thereby reducing the traction to the cornea so that these situations could facilitate spontaneous avulsion.

The etiology of spontaneous avulsion of the pterygium is unknown. The fibrovascular pterygial tissues secrete transforming growth factor β (TGF-β), a multifunctional cytokine, leading to fibroblast activation and expression of several types of matrix metalloproteinases (MMPs). These pathologies result in dissolution of hemidesmosome attachments, Bowman's layer, and the basement membrane of the cornea.^8^ We have demonstrated pterygium tissues adhere to the corneal tissues via E-cadherin and beta-catenin, which are intercellular adhesion molecules.^9^ In our present case, it is thought that spheroidal degeneration existed in broad stromal tissues, resulting in a decrease in the number of fibroblasts and microvessels and in reduced expression of the related factors. Thereby degenerative pterygial tissues may have weakened the adhesion of the pterygium to the cornea.

In this case, the total astigmatism had against-the-rule astigmatism rather than with-the-rule astigmatism before the spontaneous avulsion of the pterygium head. One of the reasons might have been presumably weak corneal astigmatism due to mild adhesion with the pterygial tissue and flattening of the cornea. In fact, the corneal astigmatism showed an astigmatic shift toward 90° after avulsion of the pterygium, and an astigmatic shift toward 180° in recurrence of the pterygium.

In addition, the pterygium is often preceded or accompanied by a pinguecula, and a previous report has shown that spheroidal degeneration is not often associated with the pterygium, but is commonly associated with the pinguecula.^10^ Therefore, in our case, the yellow-whitish elevated lesion may be a pinguecular element within the pterygium which complicates spheroidal degeneration.

Although rare, the present report has several limitations. First, the patient has received neoadjuvant chemotherapy with gemcitabine and nab-paclitaxel and postoperative adjuvant chemotherapy with S-1 because of her pancreatic head cancer. However, although these chemotherapeutic agents are broadly used worldwide, there is no previous report of pterygium avulsion caused by these agents. Therefore, it is unlikely that chemotherapy caused the pterygium avulsion. Second, we did not remove the avulsed pterygium. This is because corneal erosion was spontaneously healed after the pterygium avulsion, and the patient did not wish to receive additional treatments or any surgical interventions. For this reason, we couldn't examine histopathology to determine whether the avulsed pterygium head has a spheroidal degeneration in the present case.

In conclusion, we reported a case of spontaneous avulsion of the pterygium head thought to have been complicated by spheroidal degeneration. And it is possible that the pterygium with spheroidal degeneration may be weakly adherent and easy to avulse.

## Authorship

All listed authors meet the ICMJE criteria. We attest that all authors contributed significantly to the creation of this manuscript, each having fulfilled criteria as established by the ICMJE.

## Funding

The authors received no financial support for this research, authorship, and/or publication of this article.

## Declaration of conflicts of interest

The following authors have no financial disclosure: TT, SK, and SI.

## Statement of informed consent/patient consent

Informed consent to publish an identifiable photograph was obtained from the study participant. Written consent to publish this case has been obtained.

## Declaration of competing interest

No conflict of interest exists.
